# Electrophysiological dynamics of Chinese phonology during visual word recognition in Chinese-English bilinguals

**DOI:** 10.1038/s41598-018-25072-w

**Published:** 2018-05-02

**Authors:** Yun Wen, Ruth Filik, Walter J. B. van Heuven

**Affiliations:** 1 School of Psychology, University of Nottingham, Nottingham, UK; 2 Laboratoire de Psychologie Cognitive, Aix-Marseille Université and Centre National de la Recherche Scientifique, Marseille, France

## Abstract

Silent word reading leads to the activation of orthographic (spelling), semantic (meaning), as well as phonological (sound) information. For bilinguals, native language information can also be activated automatically when they read words in their second language. For example, when Chinese-English bilinguals read words in their second language (English), the phonology of the Chinese translations is automatically activated. Chinese phonology, however, consists of consonants and vowels (segmental) and tonal information. To what extent these two aspects of Chinese phonology are activated is yet unclear. Here, we used behavioural measures, event-related potentials and oscillatory EEG to investigate Chinese segmental and tonal activation during word recognition. Evidence of Chinese segmental activation was found when bilinguals read English words (faster responses, reduced N400, gamma-band power reduction) and when they read Chinese words (increased LPC, gamma-band power reduction). In contrast, evidence for Chinese tonal activation was only found when bilinguals read Chinese words (gamma-band power increase). Together, our converging behavioural and electrophysiological evidence indicates that Chinese segmental information is activated during English word reading, whereas both segmental and tonal information are activated during Chinese word reading. Importantly, gamma-band oscillations are modulated differently by tonal and segmental activation, suggesting independent processing of Chinese tones and segments.

## Introduction

Reading is a complex cognitive skill unique to humans. A fundamental component of this skill is the recognition of individual words. In current theories of visual word recognition, the word recognition process is affected not only by written characteristics (orthography) but also by the spoken characteristics (phonology) of target words^1^. This is supported by data obtained with alphabetical languages^2–6^ as well as non-alphabetic languages, such as Chinese^7–13^.

The activation of phonology during visual word recognition is not limited to the target language. Studies with Chinese-English bilinguals have demonstrated that the native language phonology (Chinese) is activated during second language (English) word recognition in a purely English task context^14–18^. For example, Wu and Thierry^16^ asked Chinese-English bilinguals to judge whether English word pairs were related in meaning or not (i.e., a semantic relatedness judgment task). Participants were not aware that the Chinese translations of some of the English word pairs contained phonological repetitions (e.g., *experience* - *surprise*, /**Jing1**Yan4/ - /**Jing1**Ya4/) or orthographic repetitions (e.g., *accountant* - *conference*, /Kuai4Ji4/ - /Hui4Yi4/). In their event-related potentials (ERPs) data, a reduced N400 was observed for English word pairs featuring a Chinese phonological repetition but not for pairs involving a Chinese orthographic repetition. This would indicate that Chinese phonology, rather than orthography, was activated during English visual word recognition. Phonological activation of Chinese translations as evidenced by an N400 reduction was also found in a study that manipulated the emotional valence of English word pairs^18^. Furthermore, activation of the phonology of Chinese translations was also observed during a visual shape searching task when critical English words could be entirely ignored by participants^15,17^. Taken together, these studies indicate that the phonology of Chinese translations is activated during English word recognition.

Chinese is, however, a tone language in which the phonological component of a Chinese character consists of both segments (consonants and vowels) and a lexical tone. For example, in Mandarin Chinese (henceforth Chinese), the segment Ji with a high level tone (tone 1), a rising tone (tone 2), a falling-rising tone (tone 3), and a falling tone (tone 4) can mean “chicken” (/Ji1/), “hurry” (/Ji2/), “squeeze” (/Ji3/), and “send” (/Ji4/) respectively. Note that tone is marked with numbers in Pinyin (romanisation system for Chinese phonology, indicated between slashes), whereas tone is not marked in Chinese characters. Because segment and tone are not explicitly marked in Chinese characters, grapheme-phoneme correspondences are opaque and therefore Chinese has a deep orthography. However, previous EEG (electroencephalogram) studies have found that phonology is still activated during Chinese visual word recognition. For example, in a Chinese semantic relatedness judgment experiment, Kong *et al*.^7^ found a larger P200 amplitude when a Chinese character was preceded by its homophone (e.g.,  – , /Qian4/ – /Qain4/). Such phonological repetition in Chinese has been found to be indexed by either a P200^7,13^ or an N400 effect^16,18^ and the phonological repetition stimuli used in the literature have so far always involved identical segments and tones. As far as we are aware, no studies have used EEG to investigate the activation of segment and tone separately in Chinese visual word recognition. However, a few behavioural investigations have been conducted with the colour-naming Stroop task^12,19^. For example, Li, *et al*.^19^ presented in the Stroop task colour words (e.g., /Hong2/, “red”) as well as homophones of colour words (+*Segment* +*Tone*, e.g., /**Hong2/**, “flood”), words sharing segments with colour words (+*Segment -Tone*, e.g., /**Hong**1/, “boom”), and words sharing tones with colour words (*−Segment* +*Tone*, e.g., /Ping**2**/, “bottle”). In the congruent conditions (e.g., , , ,  presented in red), the +*Segment* +*Tone*, +*Segment −Tone*, *−Segment* +*Tone* conditions all showed facilitation effects, suggesting independent activation of segment and tone. Furthermore, computational models of Chinese word processing assume *de facto* that segmental and tonal representations are independent, because they code segmental and tonal information separately^20–22^. Thus, consistent with the findings of Stroop experiments and models of Chinese word processing, we predict that segmental and tonal information play an independent role in visual word recognition in Chinese-English bilinguals.

To investigate the role of Chinese segmental and tonal activation in bilingual visual word recognition, we conducted two EEG experiments with Chinese-English bilinguals. In the first experiment, we investigated Chinese segmental and tonal activation during English word reading in a semantic relatedness judgment task with English word pairs (prime-target). The repetition of segmental and tonal information of the Chinese translations (i.e., the first character of Chinese translations for the English words) was systematically manipulated in semantically and orthographically unrelated English prime and target pairs, i.e., segmental and tonal repetition (+*Segment* +*Tone*): *media* - *rose*, /**Mei2**Ti3/ −/**Mei2**Gui4/; only segmental repetition (+*Segment −Tone*): *mirror* - *police*, /**Jing**4Zi3/ - /**Jing**3Cha2/; only tonal repetition (−*Segment* +*Tone*): *milk* - *file*, /Niu**2**Nai3/ -/Wen**2**Jian4/; no repetition (*−Segment −Tone*): *card* - *frog*, /Ka3Pian4/ - /Qing1Wa1/. In the second experiment, we investigated Chinese segmental and tonal activation during Chinese word reading in a Chinese semantic relatedness judgment task. Participants in this experiment read the Chinese translations of the English word pairs used in the first experiment. Together, these two experiments investigate indirect and direct activation of Chinese phonology during visual word recognition and aim to dissociate segmental and tonal aspects of Chinese phonology. It is important to note that Chinese phonological activation in English word reading is through translation, and that spelling-sound correspondences are opaque in Chinese. Therefore, if evidence of phonological activation is found in both experiments, it will strongly support the idea that native language phonology is an essential component of visual word recognition in the native language^23–25^ and second language^26^.

In addition to analysing ERPs, we also conducted time-frequency analyses on the EEG data to investigate event-related oscillations (EROs). An advantage of EROs is that they reveal non-phase-locked signals that conventional ERP data cannot capture^27–29^. Oscillations observed in the scalp EEG are assumed to reflect underlying neuronal synchronization^30,31^. When the power of oscillations relatively increases, it is referred to as an event-related synchronization, whereas a relative power decrease is termed an event-related desynchronization. Changes in EROs in response to linguistic manipulations have been reported in almost all major frequency bands (theta: 4–7 Hz, alpha: 8–12 Hz, lower beta: 15–20 Hz, higher beta: 20–30 Hz, gamma: above 30 Hz)^30,31^. The importance of analysing EROs in addition to ERPs is nicely illustrated in Hagoort, *et al*.^32^. This seminal study found that semantic violations and world knowledge violations modulated the N400 amplitude, whereas only world knowledge violations modulated gamma-band power. Thus, gamma activity was only modulated by world knowledge violations, but not semantic violations, which suggests that the cognitive processes involving world knowledge and semantics are separable. This finding cannot be clearly demonstrated with ERPs alone.

In sum, we investigated Chinese segmental and tonal activation during visual word recognition in Chinese-English bilinguals. If Chinese segmental and tonal information are activated independently, changes in ERPs and EROs are expected for both segmental and tonal repetition conditions. Alternatively, if only segmental or tonal information is activated, changes in ERPs and EROs would only be expected in one of these repetition conditions. Because an N400 reduction has been consistently reported when English word pairs contained a phonological repetition in the Chinese translations^14,16,18^, we focussed in our ERP analysis of the English experiment on the N400 component. In contrast, in Chinese visual word recognition the P200^7,13^ and N400^16,18^ have been found to be sensitive to phonological repetition. No previous similar word recognition studies have analysed EROs and therefore we did not have prior knowledge about which frequency bands, time windows, and electrodes may be sensitive to the effects of Chinese phonological repetition. Thus, our analyses of EROs are more exploratory and involve a data-driven approach. A behavioural impact of Chinese segmental and tonal repetition was not expected in English because no behavioural effects have been found previously^14,16,18^. For Chinese, the behavioural effects reported in the literature have so far been mixed^14,16,18^.

## Results

### Behavioural Results

A summary of the mean error rates and reaction times for the four experimental conditions in the English and Chinese experiments is presented in Table S1 of the Supplementary Material.

Responses to English word pairs with repeated Chinese segments in their Chinese translations were 14 ms faster than to English words pairs without repeated Chinese segments, *F* (1, 18) = 8.968, *p* < 0.01 η_p_^2^ = 0.333. No other effects were found in the response times (all *p*s > 0.30) and error rates (all *p*s > 0.12).

### ERP Results

The ERP results were based on the cluster-based random permutation test implemented in FieldTrip (see below for details).

#### English Experiment

**Figure 1:**
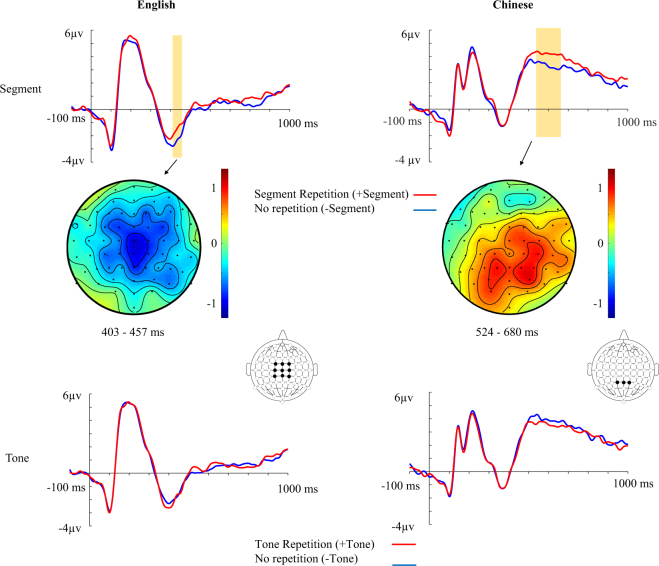
Results of ERP analyses of English (left) and Chinese (right) experiments. ERPs time-locked to the onset of target words (English experiment: averaged across 9 electrodes FC1, FC2, FCz, C1, C2, Cz, CP1, CP2, CPz, Chinese experiment: averaged across 3 electrodes, Pz, P1, P2) with topography of ERP effect.

A reduced N400 was found in the time window from 403 to 457 ms for English word pairs with segmental repetition in their Chinese translations (cluster with *p* = 0.024, see Fig. 1). There was no effect of tonal repetition or an interaction between segmental and tonal repetition.

#### Chinese Experiment

A significant effect of segmental repetition in the time window from 524 to 680 ms was found (cluster with *p* = 0.034) because Chinese words pairs with segmental repetition elicited a larger LPC (i.e., late positive component) than Chinese word pairs without segmental repetition. The difference in the LPC was found to be more pronounced over posterior electrodes (see Fig. 1). There was no effect of tonal repetition or an interaction between segmental and tonal repetition.

### Time-Frequency Results

The time-frequency results were also based on the cluster-based random permutation test implemented in FieldTrip (see below for details).

#### English Experiment

**Figure 2:**
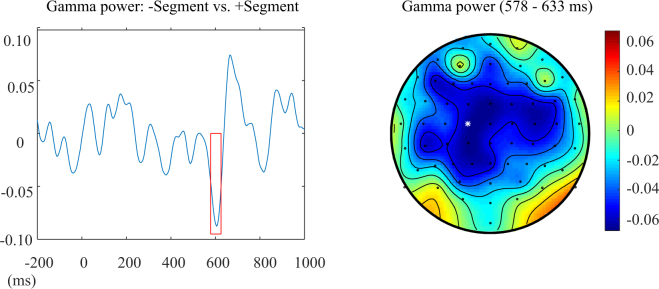
Results of time-frequency analyses for the effect of Segment in the English experiment. Gamma frequency range of the contrast +*Segment* minus *−Segment* in the C1 electrode (left); the topography of gamma frequency range from 578 to 633 ms with the white star marking the location of the C1 electrode (right). The colour scale indicates the relative power changes (0 = no difference).

Gamma power (30–60 Hz) was significantly weaker in the time window from 578 to 633 ms when segments of the Chinese translations of English word pairs were repeated than when there was no segmental repetition in the Chinese translations (cluster with *p* = 0.026). This effect was most pronounced over central electrodes (see Fig. 2). There were no gamma-band power modulations by tonal repetition, and no interaction. Additional analyses in the low frequency range (5–30 Hz) did not reveal any significant effects (see supplementary material).

#### Chinese Experiment

**Figure 3:**
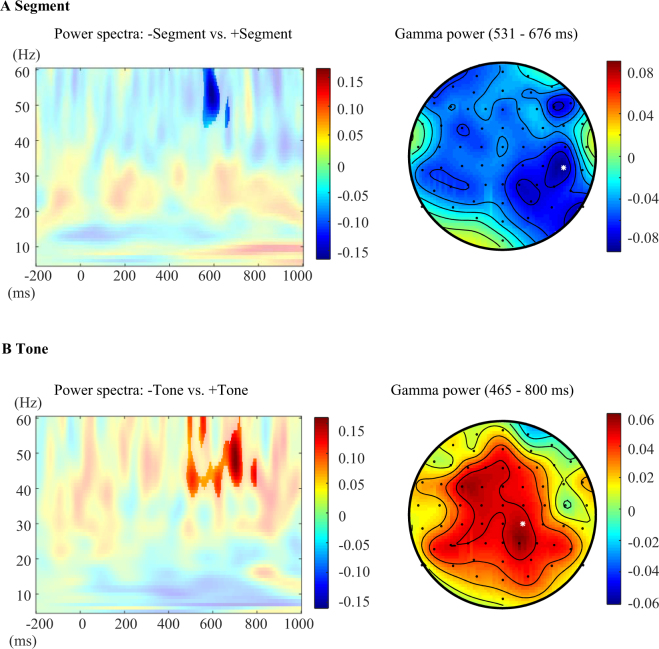
Results of time-frequency analyses in the Chinese experiment. (**A**) Time-frequency representations of the contrast +*Segment* minus *−Segment* at the CP6 electrode with a statistical mask (left); the topography of gamma frequency range from 531 to 676 ms with the white star marking the location of the CP6 electrode (right). (**B**) Time-frequency representations of the contrast +*Tone* minus *−Tone* at the CP2 electrode with a statistical mask (left); the topography of gamma frequency range from 465 to 800 ms with the white star marking the location of the CP2 electrode (right). The colour scale indicates the relative power changes (0 = no difference).

For Chinese word pairs, segmental repetition effects were found in the gamma-band power (44–60 Hz), indicating weaker effects for word pairs with repeated segments than for word pairs without repeated segments (cluster with *p* = 0.040, 531 to 676 ms). As can be seen in Fig. 3, the decreased gamma-band power for segmental repetition was widely distributed. For Chinese word pairs with repeated tones, the gamma-power (31–60 Hz) was significantly stronger in the time window from 465 to 800 ms for word pairs with the same tone than for word pairs with a different tone (cluster with *p* = 0.004). The increased gamma-band power in tonal repetition was widely distributed. No interaction was found between segmental and tonal repetition. No significant clusters were found in the low frequency range (5–30 Hz).

## Discussion

Two experiments investigated the electrophysiological dynamics of Chinese segmental (consonants and vowels) and tonal activation in Chinese-English bilinguals. In the English semantic relatedness judgement task, a hidden segmental repetition effect was observed in the behavioural data (faster responses), the ERP data (a reduced N400), and in the time-frequency data (gamma-band power reduction). These converging behavioural, ERP, and time-frequency results indicate that Chinese segmental information (consonants and vowels) of Chinese translations is activated during English word reading. In the Chinese experiment, the segmental repetition effect was observed in the ERP data (an increased LPC) and the time-frequency data (gamma-band power reduction). In addition, we found a tonal repetition effect in the time-frequency data (gamma-band power increase). Taken together, the combined results of the two experiments indicate that when Chinese-English bilinguals read English words, the segmental information of Chinese translations is activated, whereas when they read Chinese words both segmental and tonal information are activated.

We did not expect to find behavioural effects of segmental repetition in the English experiment because previous studies using the same paradigm did not find significant behavioural effects^14,16,18^. One possible explanation for our behavioural finding is that our experiment included more trials with segmental repetition than previous studies (60 trials for + *Segment* +*Tone* plus 60 trials for +*Segment −Tone* vs. 45 trials for +*Segment* +*Tone* in Wu and Thierry^16^). Using more trials can reduce the noise in the data and therefore increase the chance to detect an effect.

The N400 reduction observed in the English experiment is consistent with previous studies revealing activation of the first language during second language reading^14–18,33–36^. These findings support the idea that visual word recognition in bilinguals involves non-selective lexical access^26,37^. Importantly, because the N400 reduction was found for hidden segmental repetition but not tonal repetition, it suggests that the hidden phonological repetition observed in previous studies^14,16,18^ is likely driven by segmental repetition in the Chinese translations of English word pairs. Furthermore, the N400 reduction elicited by the hidden segmental repetition supports previous suggestions that the N400 component is an index of phonological priming^2,5^. Given that the N400 component can also reflect semantic processing of English words in the semantic relatedness judgement task, the observed N400 reduction indicates that Chinese phonology may impact the on-going semantic processing of English words.

An unexpected finding of the present study is that the ERP effects of segmental repetition were different in English and Chinese. The N400 effect in English occurred in the time window that has been associated with lexical processing, whereas the LPC effect in Chinese occurred at the decision stage^33,38^. The LPC is assumed to reflect conscious conflict resolution^33,38^ that might specifically be needed when the Chinese word pairs had overt segmental repetition. This conscious conflict resolution may abolish any behavioural facilitation effects due to segmental repetition. Therefore, the presence of the LPC in the Chinese experiment and its absence in the English experiment may explain why behavioural effects of segmental repetition were only observed in English.

The ERP effects of segmental repetition in Chinese occurred later than in previous studies investigating phonological activation in Chinese word recognition. However, these studies have focussed mainly on the contrast between +*Segment* +*Tone* and *−Segment −Tone*, and reported early ERP effects (e.g., N400 effect^16,18^ or a P200 effect^7,13^). To compare our data with previous studies, a new analysis of the Chinese experiment was conducted to directly compare +*Segment* +*Tone* and *−Segment −Tone*. A significant P200 reduction was found for +*Segment* +*Tone* (see Figure S6 in the Supplementary Material), suggesting that Chinese phonological activation impacted ERP responses in an early time window. This P200 effect resembled the patterns reported in Thierry and Wu^14^, which showed a reduction of the P200 in Chinese word pairs containing orthographic and phonological repetitions (e.g., [**Hou3**Che1] –[**Huo3**Tui3]). However, it is unclear why the phonological repetition (+*Segment* +*Tone*) elicited an enhanced P200 in previous studies^7,13^, whereas the current data showed a reduced P200. Although the P200 component has been associated with lexical form overlap^8,38^, the exact mechanisms underlying the decrease and increase of the P200 remains unclear (for a discussion, see^33^). One possible explanation for the discrepancy of the P200 modulations is that two-character Chinese words were presented in the present study in contrast to single characters used in previous studies^7,13^. In other words, different P200 modulations may indicate that the phonological repetition in two-character words is different from that in single characters.

Time-frequency analyses have so far only been conducted in a few bilingual studies, such as studies involving sentence reading^39,40^, word production^41^, and language learning^42–44^. As far as we are aware, the current study is the first to use oscillatory EEG data to investigate bilingual visual word recognition. Our key finding is that gamma-band power is reduced by segmental repetition of Chinese translations during an English semantic relatedness task. There is unfortunately no one-to-one mapping between cognitive functions and gamma band power^45^. However, one way to interpret our findings is to look at the findings of previous studies investigating repetition. Various types of stimulus repetition (e.g., words, drawings, pictures) have been reported to cause a reduction of neural activity, which is often referred to as repetition suppression^46–51^. Repetition suppression commonly correlates with decreases in haemodynamic responses in fMRI/PET studies and in gamma-band power in EEG studies^50^. Although the exact neural mechanism underlying repetition suppression is unclear, it can generally be understood as greater neural efficiency in processing repeated stimuli^50^. For example, in a masked priming experiment with briefly presented primes (i.e., 30 ms)^52^, gamma-band power was reduced for homophone primes (e.g., *pair* - *pear*) compared to control primes. Matsumoto and Iidaka^52^ interpreted this decrease in gamma-band power as repetition suppression, suggesting that repetition of one stimulus feature (phonology) is able to reduce neural activity. The reduced gamma power elicited by segmental repetition in both the Chinese translations of English word pairs in the English experiment and the Chinese words in the Chinese experiment is consistent with this interpretation. The gamma power reduction was not associated with the modulation of specific ERP components. This is because conventional ERP data cannot capture non-phase-locked signals (unlike EROs), and frequencies above 15 Hz are usually non-phase-locked^27–29^. Furthermore, low-pass filters were applied in the ERP analyses as is common in many language studies (e.g., 30 Hz in the present study and 25 Hz in Wu and Thierry). Therefore, signals from the gamma frequency range (>30 Hz) were largely attenuated.

Another important finding of the current study is that tonal repetition increased gamma-band power in the Chinese experiment. Because tonal repetition effects were not found in the behavioural and ERP data, it turns out to be crucial to analyse both phase and non-phase locked EEG signals when studying the role of phonology in Chinese word processing. Our study provides, as far as we are aware, the first electrophysiological evidence of tonal activation when native Chinese speakers read two-character Chinese words. Previous investigations of tonal activation during Chinese word reading have mainly focussed on single Chinese characters^19,53,54^. For example, using a visual odd-ball paradigm, two ERP studies^53,54^ have reported a visual mismatch negativity elicited by characters with a deviant tone (e.g., /Yi1/) presented amongst characters with a standard tone (e.g., , , , , /Yi4/), suggesting that tonal information is quickly activated. However, single character recognition may not provide the complete picture of tonal activation during Chinese word recognition because there are much fewer one-character than two-character Chinese words (e.g., 5,321 vs. 45,871 in SUBTLEX-CH^55^). It is not yet clear why gamma-band power increased in response to tonal repetition but decreased in response to segmental repetition. The different direction of gamma-band modulation for tonal and segmental repetition may be a sign of functional dissociation between tonal and segmental information. However, this explanation seems at odds with the idea that a gamma-band decrease in response to segmental repetition is the neural consequence of repetition suppression. Alternatively, a gamma-band increase elicited by tonal repetition could be interpreted as repetition enhancement^56^. Stimuli-related repetition enhancement has in fact been observed in other studies^46,49,56^, and the contrast between repetition suppression and enhancement might be related to differences in the types of repeated stimuli. For example, gamma-band power has been found to increase when pseudowords were repeated, whereas gamma-band power reduction has been found for word repetition^49^.

The different patterns of gamma-band power modulation in segmental and tonal repetition could be related to the distribution of repeated segments and tones. There are in Chinese 404 different syllables^57^, whereas there are only four different tones. Thus, segmental repetition may be more salient than tonal repetition. Furthermore, native Chinese speakers often rely on Pinyin to enter Chinese characters on computers and mobile phones. Because only segmental information is required for typing Pinyin, segmental information may be activated more often than tonal information. Thus, native Chinese speakers may be more sensitive to segmental repetition than tonal repetition. However, these interpretations are speculative, and therefore further research is needed to examine why gamma band modulations are affected differently by segmental and tonal repetition.

In sum, we showed that brain oscillations are influenced by Chinese phonological activation during visual word recognition in Chinese-English bilinguals. We obtained converging behavioural and electrophysiological evidence that segmental information of Chinese translations is activated during English word reading, indicating that previous phonological repetition effects observed in the literature are likely due to specifically Chinese segmental repetition. In contrast, both segmental and tonal information are activated during Chinese word recognition. Importantly, tonal repetition effects were only found in the time-frequency data, illustrating that oscillatory EEG data are crucial in detecting the activation of tonal features of words. The present study advances our understanding of the role of Chinese segmental and tonal information in visual word recognition and highlights the importance of brain oscillations in studying language processing.

## Methods

### Participants

In total, 64 Chinese-English bilinguals participated (32 in each experiment) and they were all paid for their participation. Participants were all native Mandarin Chinese speakers who learnt Chinese from birth, and they were undergraduate or postgraduate university students studying in Nottingham. They all met the minimum English language entry requirements to study at the University of Nottingham (IELTS 6.0 for undergraduates and 6.5 for postgraduates). The details of participants’ language background are summarized in Table 1. All participants had normal or corrected-to-normal vision and had no history of neurological or psychiatric impairment. According to an adapted version of Coren’s handedness questionnaire^58^ (available at http://faculty.washington.edu/chudler/rltablen.html), all participants were right-handed. For the English experiment, one participant was excluded due to high error rates (>25%), and one participant was excluded due to excessive eye blinks. Because the data were recorded during a hot summer in a lab without air-conditioning, an additional 11 participants in the English experiment and nine participants in the Chinese experiment were excluded from the analyses due to slow drifts. A minimum of 40 epochs were required for each condition and the analyses were based on 19 participants in the English experiment and 23 participants in the Chinese experiment.

**Table 1:** Summary of the participants’ language background.

	Mean (SD)	
	English experiment	Chinese experiment
Age (years)	23.1 (2.7)	24.4 (2.9)
Age exposed to formal English education	10.1 (2.0)	10.3 (2.3)
Time studies English (years)	13.1 (2.3)	14.1 (2.8)
English immersion experience (months)	17.4 (13.9)	21.1 (19.9)
LexTALE test score	58.2 (9.0)	57.2 (7.0)
Self-rated English Reading ability	5.0 (0.7)	5.0 (0.7)

*Note*. LexTALE^61^ is an English vocabulary test; subjective reading ability was rated on a 7-point scale (1 = very poor, 7 = native-like).

### Materials and Design

Using the data from the English-Chinese translation norming study reported in Wen and van Heuven^59^, 240 experimental English words pairs with similar word frequency, length, and concreteness were selected to create four experimental conditions in the English experiment: +*Segment* +*Tone*, +*Segment −Tone*, *−Segment* +*Tone* and *−Segment −Tone*. For the Chinese experiment, the materials and design were identical to the English experiment except that participants read the Chinese translations of the English word pairs. Examples of stimuli are presented in Table S2 of the Supplementary Material. All critical 240 word pairs were unrelated in meaning, whereas 60 filler pairs were semantically related.

### Procedure

The experiments were approved by the Ethics Committee of the School of Psychology, University of Nottingham. The experiments were performed in accordance with approved guidelines and regulations. All participants gave their written informed consent before the experiment started. Participants were tested individually in a dimly-lit shielded experimental room. Each participant received a unique random presentation order of the stimuli. Stimuli were presented on a CRT monitor (17″ Liyama Vision Master 400, 1024 × 768 pixels, 85 Hz) controlled by PsychoPy^60^. Each trial began with a fixation cross, presented for 500 ms followed by a 300 ms black screen. Next, the prime word (in lowercase for the English experiment) was presented for 500 ms, followed by a variable interstimulus interval of 500, 600 or 700 ms^14,16^ after which the target word (in uppercase for the English experiment) with a fixed presentation time of 1000 ms was presented. Participants had to decide whether the presented word pairs (prime-target) were related in meaning or not by pressing buttons on a Cedrus response box attached to the computer. They were instructed to perform the task as quickly and as accurately as possible. If participants did not respond during the presentation of the target, a blank screen would appear for up to a maximum of 2000 ms or until the participants responded. Response sides were counterbalanced between participants. The inter-trial interval was set at 1000 ms.

Participants in the English experiment made semantic relatedness judgments to 300 English word pairs, and participants in the Chinese experiment performed the same task to Chinese translations of the 300 English word pairs. Prior to the experiment, a practice block of 15 trials was used to familiarize the participants with the procedure. Participants were instructed to minimize blinks, eye-movements, and body movements during stimuli presentation. After the semantic relatedness judgment task, participants completed an English vocabulary test^61^ and a language history questionnaire. The entire session for each experiment lasted about two hours.

### EEG Recording and Preprocessing

The electroencephalogram (EEG) was recorded continuously at a sample rate of 1024 Hz using a Biosemi ActiveTwo system with 64 Ag/AgCl active electrodes placed according to the 10/20 system. Two additional electrodes placed close to Pz, namely, the Common Mode Sense [CMS] active electrode and the Driven Right Leg [DRL] passive electrode, were used for online referencing (for detailed explanations, see^62,63^). In addition to the 66 electrodes on the scalp, seven external electrodes were applied: two over left and right mastoids for off-line re-referencing, two at the outer canthus of each eye, two below each eye and one above the left eye for monitoring eye blinks and movements. For each measurement site, the voltage offset from the CMS was below 25 mV.

Data were preprocessed and analysed using FieldTrip^64^. The continuous EEG was filtered offline with a high-pass filter set at 0.1 Hz. The EEG data were then re-referenced off-line to the averaged mastoids and segmented in epochs of 3000 ms beginning 1000 ms prior to the onset of the second word (a long epoch is required in time-frequency analyses, because the value of the power spectrum at a given time point is a weighted average of values within a certain time window centred at that point, see^27,28^). Trials with incorrect responses or RTs less than 300 ms or more than 2,500 ms were discarded (2.8% of the data). Epochs contaminated by drifts and muscle activity were first manually dismissed (8.4% of the data, mostly due to drift), and eye-blinks and eye movements were corrected using an independent component analysis (ICA) algorithm^65^. The epochs with potentials greater than 100 μV were automatically rejected (16.0% of the data). For the data of each participant to be included in the analysis, a minimum of 40 epochs were required for each condition. The preprocessed data were then used for the ERP analysis and the time-frequency analysis.

### ERP Analysis

The clean EEG data were re-segmented in epochs of 1100 ms beginning 100 ms prior to the onset of the second word. Baseline correction was applied, using the interval from −100 to 0 msec. The epochs were low-pass filtered at 30 Hz. Finally, the average of the ERPs was computed for each participant in each of the experimental conditions.

### Time-frequency Analysis

The preprocessed data were used for the time-frequency analyses. The power spectra were computed for all single trials using complex Morlet wavelets^66^. A wavelet of seven-cycle width was used, with frequencies increasing from 5 to 60 Hz, in 1-Hz steps. The individual trial power spectra were then averaged across trials for each condition and participant. The powers obtained were expressed as a percentage change relative to the power in a baseline interval from 300 to 100 ms before the target words.

### Statistical Analysis

For the behavioural data, a 2 (+Segment vs. −Segment) × 2 (+Tone vs. −Tone) repeated measures ANOVA was run for both reaction times and error rates.

The ERP data were analysed using the cluster-based random permutation test as implemented in FieldTrip (for details see^67^). The ERP data analysis of the English experiment focused on the N400 component^14,16,18^, and for the analyses nine electrodes were selected (FC1, FC2, FCz, C1, C2, Cz, CP1, CP2, CPz), which showed maximal effects in previous studies^14,16,18^. Thus, a cluster-based random permutation test was applied on an a-priori region of interest from 350 ms to 500 ms post-stimulus for the main effect of Segment and Tone and their interaction. The focus of the ERP analysis in the Chinese experiment was the P200 component^7,13^, the N400 component^14,16,18^ and the LPC component^33,38^ (see Supplementary Material for more discussion). To capture these three components, the cluster-based random permutation test was applied from 150 ms to 800 ms post-stimulus on all 64 electrodes.

For the time-frequency data, power in percentage change relative to baseline was tested statistically using the cluster-based random permutation test implemented in FieldTrip. We did not have prior knowledge about which frequency bands, time windows, and electrodes would be sensitive to the effect of Chinese phonological activation during English word reading. Therefore, the time-frequency results from the Chinese experiment were used to guide the time-frequency analysis of the English experiment. For the Chinese experiment, cluster-based random permutation tests were separately conducted for low- (5–30 Hz) and high-(30–60 Hz) frequency ranges^68,69^ from 200 ms to 800 ms post-stimulus on the main effects of Segment and Tone and their interaction. Therefore, cluster-based random permutation tests were conducted over each frequency, time and electrode. Because significant changes were found in the gamma-band power (30–60 Hz) approximately 400–800 ms post-stimulus, the gamma-band power (30–60 Hz) was first averaged for each electrode and each condition in the English experiment to increase the sensitivity of the permutation test^67^. For the main effects of Segment and Tone and their interaction, a cluster-based random permutation test was then conducted for the averaged gamma-band power from 400 ms to 800 ms post-stimulus. Therefore, cluster-based random permutation tests in the English experiment were conducted over time and electrode.

### Data availability

The datasets generated during and/or analysed during the current study are available from the corresponding author on reasonable request.

## Electronic supplementary material

Supplementary Material
