# Supplementary material for: Electrophysiological dynamics of Chinese phonology during visual word recognition in Chinese-English bilinguals
Source: Sci Rep. 2018 May 2;8:6869. doi: 10.1038/s41598-018-25072-w (PMC5931991; doi:10.1038/s41598-018-25072-w)
Supplement: Supplementary file 1 — Supplementary Material [file 41598_2018_25072_MOESM1_ESM.pdf]

Supplementary Material

Electrophysiological dynamics of Chinese phonology during visual word recognition in Chinese-English bilinguals

Yun Wen

Ruth Filik

Walter J. B. van Heuven

Results

Behavioural Results

Table S1 Mean error rates and reaction times (with SD in brackets) of English and Chinese Experiments

	+Segment +Tone	*+Segment -Tone*	*-Segment +Tone*	*-Segment -Tone*
**English Experiment**				
Error Rates (%)	2.11	2.28	1.58	2.11
	(2.14)	(1.59)	(1.52)	(2.60)
Reaction Time (ms)	966	947	967	974
	(247)	(245)	(248)	(250)
**Chinese Experiment**				
Error Rates (%)	1.00	1.01	1.01	1.81
	(1.93)	(2.23)	(2.23)	(3.55)
Reaction Time (ms)	643	642	643	637
	(130)	(135)	(142)	(137)

ERP Results

Figure S1 Results of the permutation test for segmental repetition in the English experiment (ERP data)

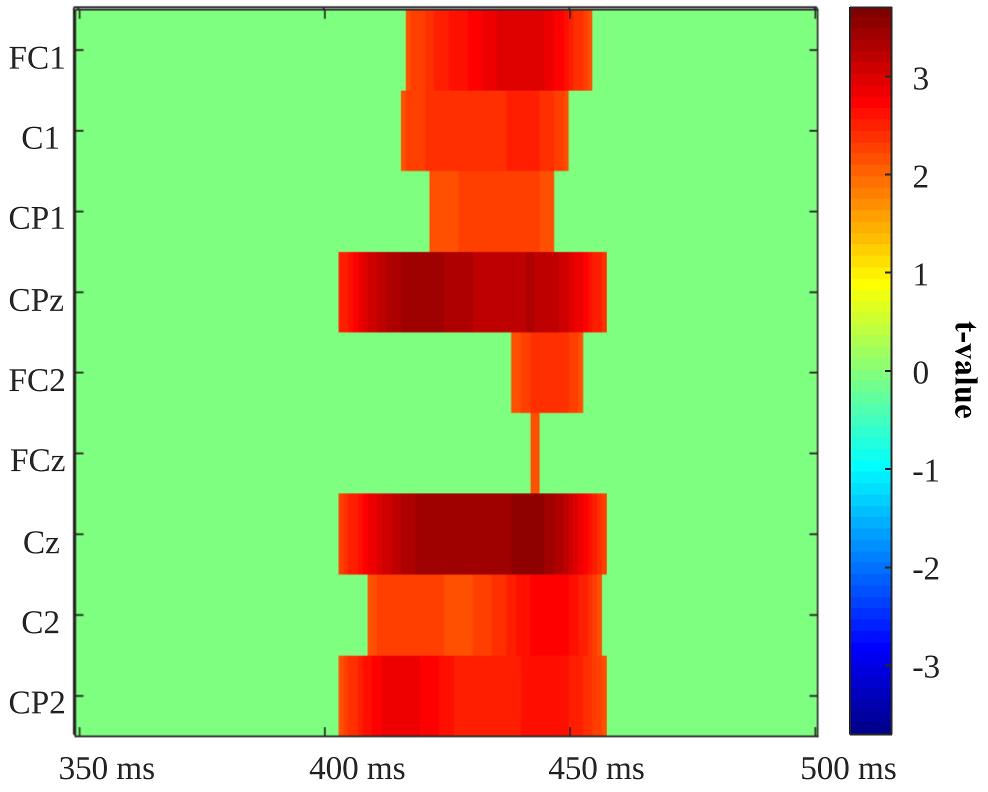

Figure S2 Results of the permutation test for segmental repetition in the Chinese experiment (ERP data)

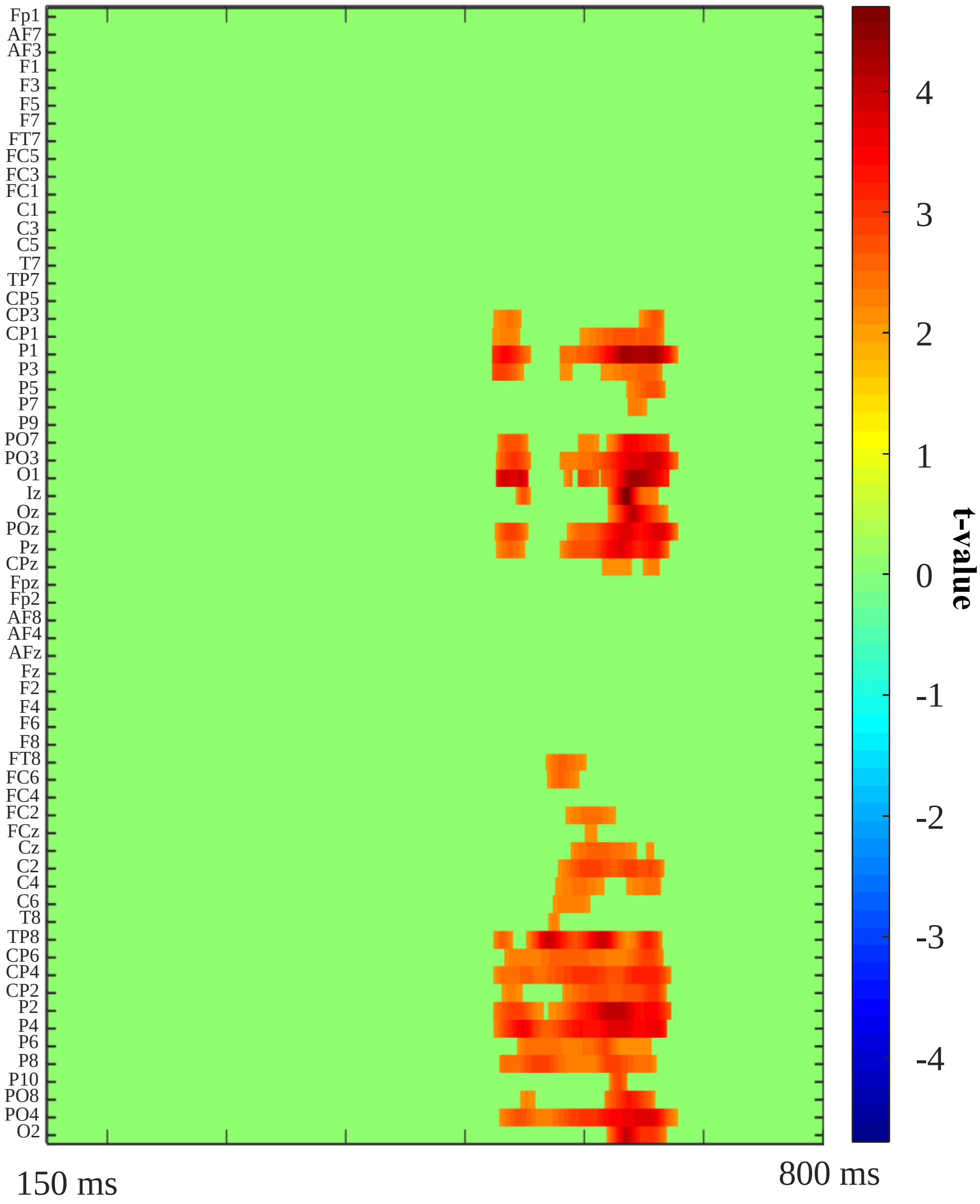

Time-frequency Results

Although our analysis of the English experiment only focused on the gamma frequency range, additional analyses were conducted in the low frequency range (5-30 Hz) as suggested by reviewers. In line with the analysis of Chinese experiment, theses cluster-based permutation tests for low frequency range included three dimensions (frequency, time, electrodes). No significant effects were found.

Figure S3 Results of the permutation test for segmental repetition in the English experiment (time-frequency data)

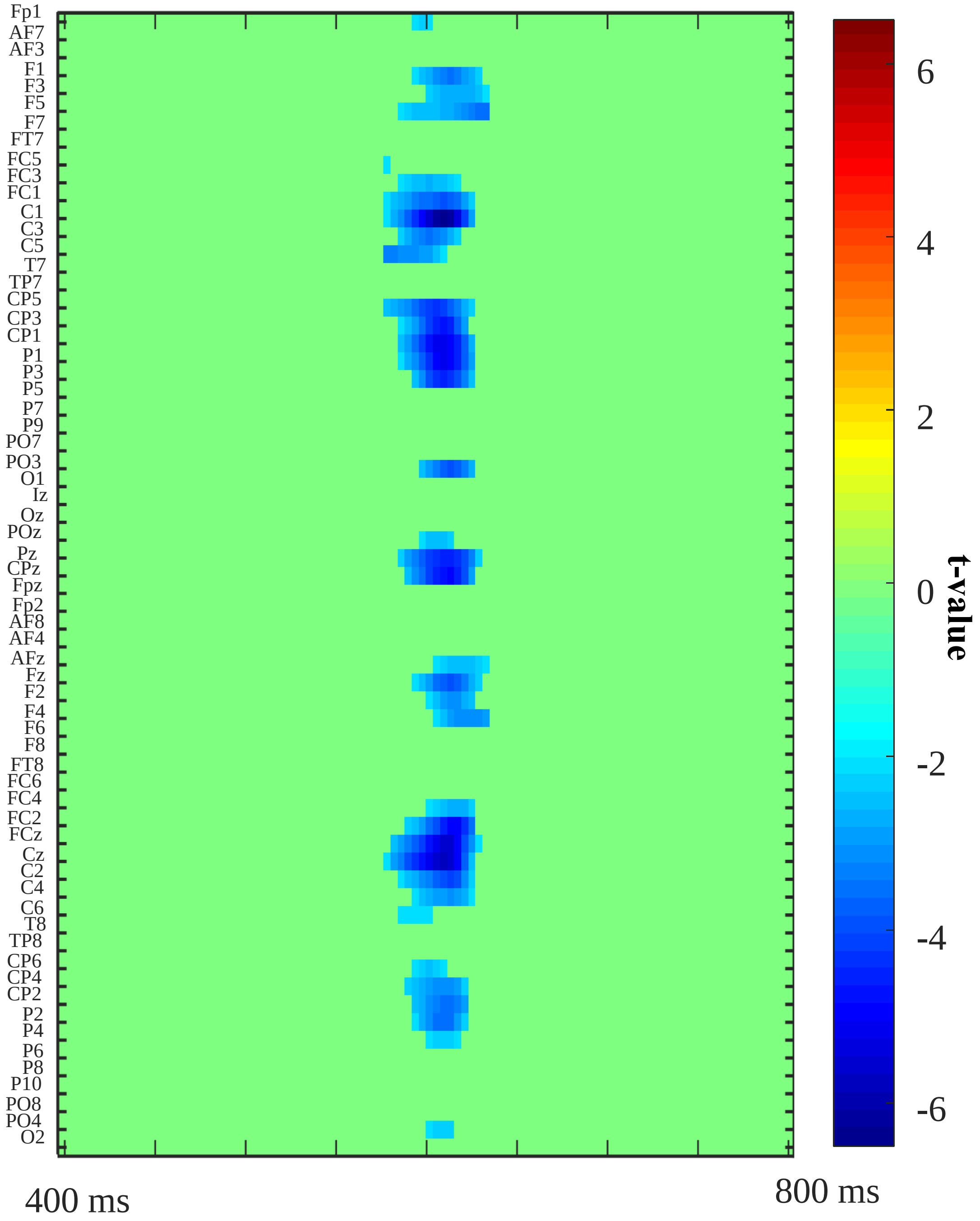

Figure S4 Results of the permutation test for segmental repetition in the Chinese experiment (time-frequency data)

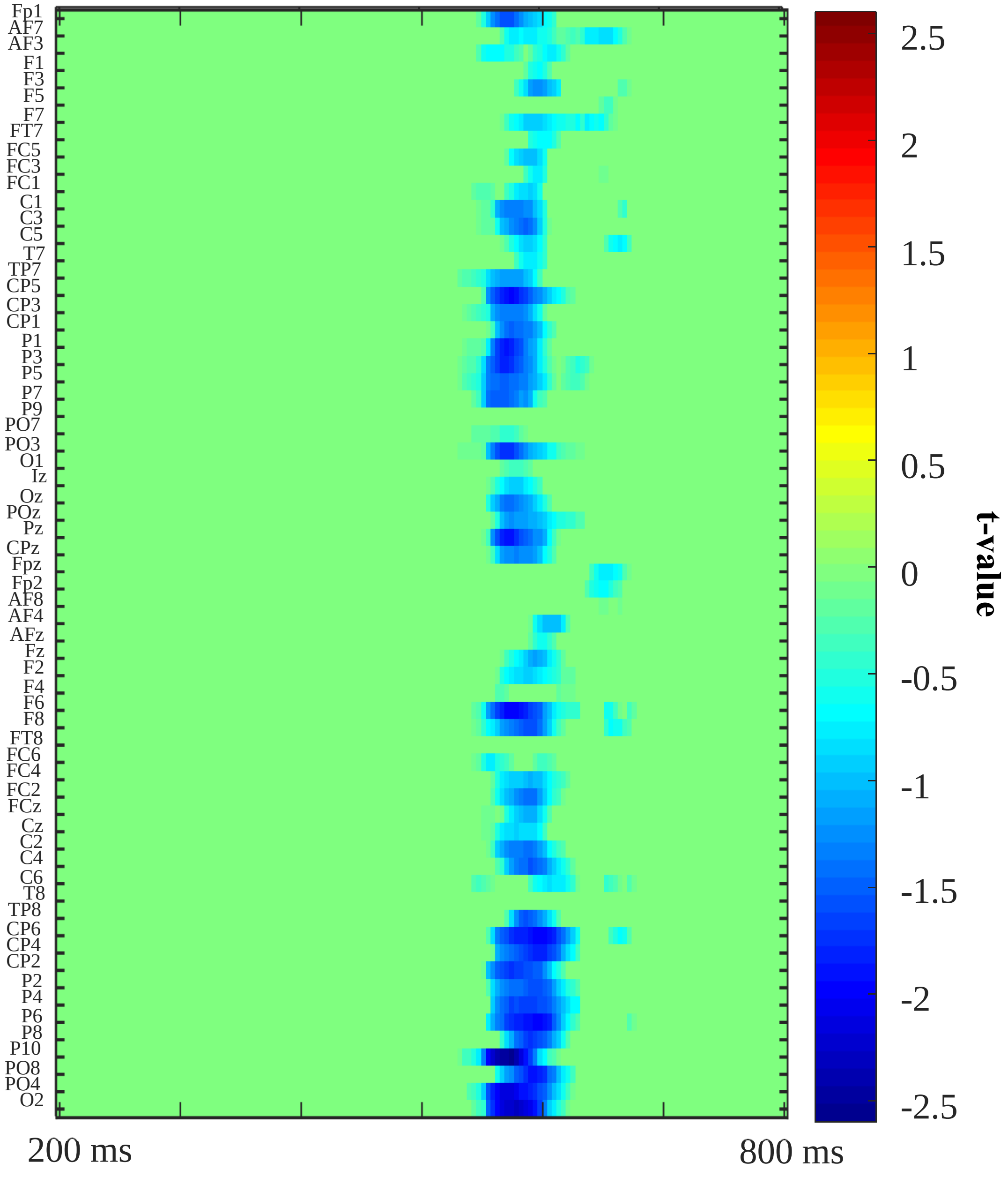

Figure S5 Results of the permutation test for tonal repetition in the Chinese experiment (time-frequency data)

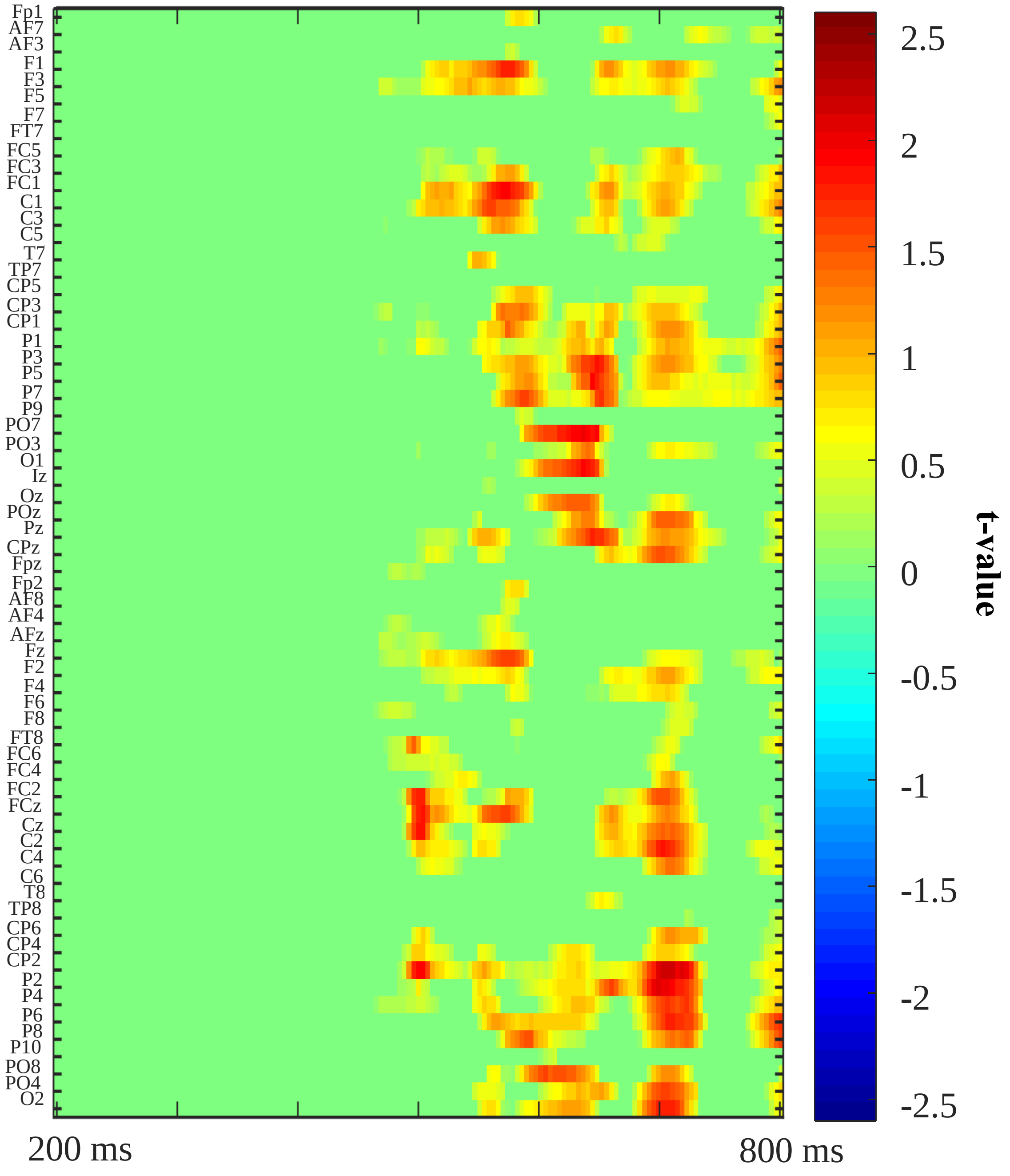

Discussion

The cluster-based permutation test revealed a significant difference between *+Segment +Tone* and *-Segment -Tone* (cluster with p = .030, 180 to 250 ms, see Figure S6)

Figure S6. Results of ERP analysis of *-Segment -Tone* vs. *+Segment +Tone*. ERPs time-locked to the onset of target words averaged across 3 electrodes (FC1, FC2, FCz, all shaded in the schematic head) on the scalp with topography of ERP effect (-*Segment -Tone* minus *+Segment +Tone*)

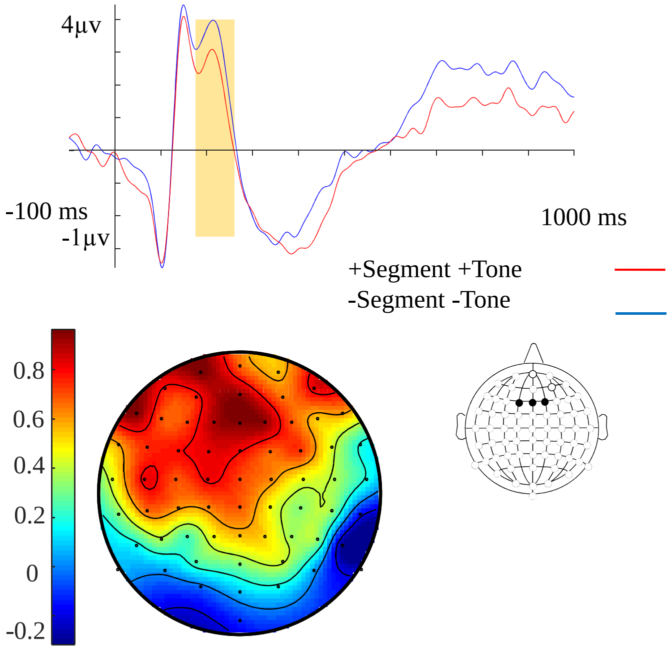

Methods

Materials and Design

Table S2 Experimental design and stimulus examples in English and Chinese Experiments

	**Experimental Trials (Semantically Unrelated)**								**Filler Trials (Semantically Related)**	
	+Segment +Tone		*+Segment -Tone*		*- Segment +Tone*		*-Segment -Tone*			
English	media -	rose	mirror -	police	milk -	file	card -	frog	hobby	interest
Chinese Translation	媒体	玫瑰	镜子	警察	牛奶	文件	卡片	青蛙	爱好	兴趣
Pinyin	***Mei2***Ti3	***Mei2***Gui4	***Jing***4JZi3	***Jing***3Cha2	Niu***2***Nai3	Wen***2***Jian4	Ka3Pian4	Qing1Wa1	Ai4Hao4	Xing4Qu4
Semantic Scores (English)	1.24(0.21)		1.21(0.24)		1.23(0.25)		1.32(0.32)		4.34(0.24)	
Semantic Scores (Chinese)	1.19(0.19)		1.16(0.20)		1.15(0.19)		1.21(0.24)		4.36(0.26)	
Word Length	5.38(1.57)	5.77(1.58)	6.13(1.63)	5.73(1.51)	6.08(1.85)	6.10(1.60)	6.37(1.92)	5.75(1.71)	6.15(2.15)	5.72(1.90)
English Frequency	4.62(0.66)	4.67(0.57)	4.49(0.55)	4.52(0.60)	4.47(0.57)	4.45(0.60)	4.41(0.65)	4.56(0.48)	4.54(0.54)	4.80(0.56)
Chinese Frequency	4.49(0.64)	4.58(0.61)	4.40(0.63)	4.47(0.70)	4.47(0.50)	4.48(0.66)	4.48(0.45)	4.52(0.53)	4.31(0.67)	4.15(0.68)
Concreteness	3.94(1.00)	3.59(1.07)	3.86(1.06)	3.87(1.00)	3.99(0.98)	3.75(1.08)	3.70(1.05)	3.88(1.06)	3.90(1.08)	4.07(0.98)

Note. Pinyin is a romanisation system for Chinese phonology. In Pinyin numbers refer to the Chinese tone. There are four different tones in Mandarin Chinese: high level tone (1), rising tone (2), falling-rising tone (3) and falling tone (4). The English frequency is based on SUBTLEX-UK Zipf values (van Heuven, Mandera, Keuleers, & Brysbaert, 2014) and Chinese frequency is based on SUBTLEX-CH Zipf values (Cai & Brysbaert, 2010). Concreteness score is based on Brysbaert, Warriner, and Kuperman (2014). Semantic Scores of English word pairs and their Chinese equivalent pairs were obtained using a 5-point semantic rating study with a different group of 20 Chinese-English bilinguals to make sure that all the critical word pairs are not related in meaning.

ERP Analysis

Previous studies have consistently reported an N400 reduction when English word pairs contained Chinese phonological repetition (Thierry & Wu, 2007; Wu & Thierry, 2010, 2012a). Thus, we had a-priori knowledge of the time-window. Furthermore, we selected electrodes which showed maximal effects in previous studies (Thierry & Wu, 2007; Wu & Thierry, 2010, 2012a). As indicated in Maris and Oostenveld (2007) and discussed in FieldTrip's on-line tutorial, cluster-based permutation tests can be conducted with a-priori selected channels and latency to increase the sensitivity. Therefore, for the English experiment, we conducted cluster-based permutation tests on a-priori selected channels and time window.

We also conducted traditional AVOVA analyses with the data of the English experiment. We first used the mean global field power across all electrodes in all semantically unrelated conditions (*+Segment +Tone*, *+Segment -Tone*, *-Segment +Tone*, *-Segment -Tone*) to detect the latency of the peak amplitude in the N400 window from 350 ms to 500 ms. The peak latency was 392 ms. Next, the mean amplitude of the time window which extended ±25 ms surrounding the peak (367 ms to 417 ms) was calculated. The mean amplitude of the next 50-ms time window (417 ms to 467 ms) was also calculated. A 2 (Segment: *+Segment* vs. *-Segment*) × 2 (Tone: *+Tone* vs. *-Tone*) × 9 (Electrodes: FC1, FC2, FCz, C1, C2, Cz, CP1, CP2, CPz) repeated measures ANOVA was conducted for the mean amplitudes in both time windows. This approach aims to capture the fine-grained changes of the N400 component, which is analogous to using pairwise t-tests for each sampling point in previous studies (e.g., Thierry & Wu, 2007; Wu & Thierry, 2010). Between 367 - 417 ms, analyses of the mean amplitudes revealed no significant effects of Segment, F (1,18) = 2.409, p = .138, and Tone, F (1,18) = 2.234, p = .152, and no interaction between Segment and Tone $F < 1$, $p > .50$. However, between 417 -

467 ms, the analysis of the mean amplitudes revealed a significant effect of Segment, F (1,18) = 6.099, p = .024, indicating a reduced N400 for segmental repetition. Thus, the results of AVOVA analyses are very similar to the cluster-based permutation tests.

For the Chinese experiment, we tried to identify the critical time-window for the P200 and N400 components using the mean global field power (Lehmann, 1987; Lehmann & Skrandies, 1980; Picton et al., 2000). However, using the mean global field power measured across all electrodes and all unrelated conditions (*+Segment +Tone*, *+Segment -Tone*, *-Segment +Tone*, *-Segment -Tone*), the peaks for 100-250 ms and 350-500 were detected at 148 ms and 459 ms respectively. The latencies of the peaks were unexpected because the 148-ms peak was relatively early for the P200 component and the 459-ms peak was relatively late for the N400 component. Based on the latencies of these peaks, it is very likely that the P200 component temporally overlapped with an early component and that the N400 component temporally overlapped with a later component. Although the mean global field power is an unbiased measure of electrical signal amplitude across the scalp, it may fail to distinguish peaks of temporally overlapping components (Dien, 2012). To further investigate the issue of overlapping components in our data, the ERP waveforms were averaged across all unrelated conditions (*+Segment +Tone*, *+Segment -Tone*, *-Segment +Tone*, *-Segment -Tone*). As can be seen in Figure S7, the P200 component is preceded by the N170 component and the N400 component is followed by the LPC component. Because the patterns in the ERP data were more complex than expected and different from previous studies, the findings of previous studies were not very helpful in guiding our analysis. Therefore, we decided to include the whole time window (200-800 ms) and all electrodes in the cluster-based permutation analysis of the Chinese experiment.

Figure S7. ERPs time-locked to the onset of target words in the Chinese experiment average across all the unrelated conditions (+*Segment* +*Tone*, +*Segment* -*Tone*, -*Segment* +*Tone*, -*Segment* -*Tone*) at the Cz electrode with the schematic head depicting the location of Cz.

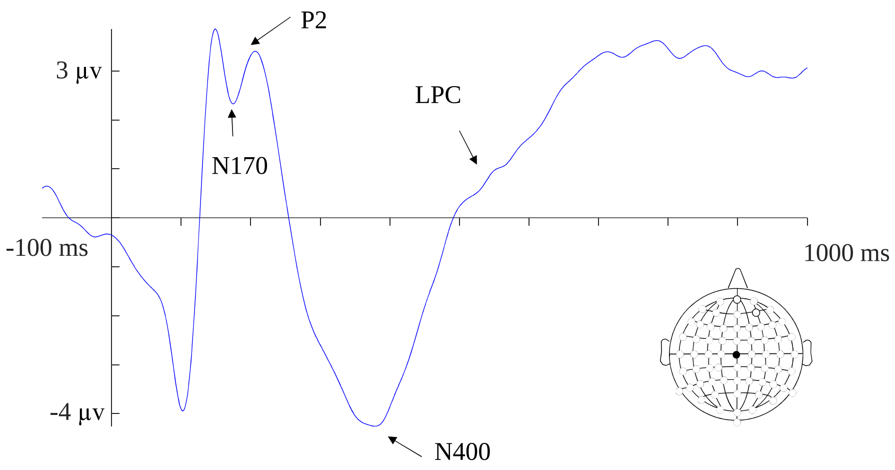

References

Brysbaert, M., Warriner, A. B., & Kuperman, V. (2014). Concreteness ratings for 40 thousand generally known English word lemmas. *Behavior Research Methods, 46*(3), 904-911. doi: 10.3758/s13428-013-0403-5

Cai, Q., & Brysbaert, M. (2010). SUBTLEX-CH: Chinese word and character frequencies based on film subtitles. *PLoS ONE, 5*(6), e10729. doi: 10.1371/journal.pone.0010729

Dien, J. (2012). Applying principal components analysis to event-related potentials: A Tutorial. *Developmental Neuropsychology, 37*(6), 497-517. doi: 10.1080/87565641.2012.697503

Lehmann, D. (1987). Principles of spatial analysis. In A. S. Gevins & A. Rémond (Eds.), *Handbook of electroencephalography and clinical neurophysiology: Revised series, Vol. 1. Analysis of electrical and magnetic signals* (pp. 390-354). Amsterdam: Elsevier.

Lehmann, D., & Skrandies, W. (1980). Reference-free identification of components of checkerboard-evoked multichannel potential fields. *Electroencephalography and Clinical Neurophysiology, 48*(6), 609-621. doi: 10.1016/0013-4694(80)90419-8

Picton, T. W., Bentin, S., Berg, P., Donchin, E., Hillyard, S. A., Johnson, R., . . . Rugg, M. D. (2000). Guidelines for using human event-related potentials to study cognition: recording standards and publication criteria. *Psychophysiology, 37*(02), 127-152. doi: 10.1111/1469-8986.3720127

van Heuven, W. J. B., Mandera, P., Keuleers, E., & Brysbaert, M. (2014). SUBTLEX-UK: A new and improved word frequency database for British English. *The*

Quarterly Journal of Experimental Psychology, 67(6), 1176-1190. doi: 10.1080/17470218.2013.850521
